# Coinheritance of Hypertrophic and Arrhythmogenic Cardiomyopathy Variants in a Patient With Hypertrophic Cardiomyopathy

**DOI:** 10.1016/j.jaccas.2024.102646

**Published:** 2024-11-20

**Authors:** Yi Siang Lee, Chee Jian Pua, Yasmin Bylstra, Saumya Shekhar Jamuar, Iswaree Devi Balakrishnan

**Affiliations:** a Yong Loo Lin School of Medicine, National University of Singapore, Singapore; b National Heart Research Institute Singapore, National Heart Centre Singapore; c SingHealth Duke-NUS Institute of Precision Medicine, Singapore; d SingHealth Duke-NUS Genomic Medicine Centre, Duke NUS Medical School, Singapore; e Genetics Service, KK Women’s and Children’s Hospital, Singapore; f Department of Cardiology, National Heart Centre Singapore, Singapore

**Keywords:** coinheritance, distinct cardiomyopathies, pathogenic variants

## Abstract

Hypertrophic cardiomyopathy (HCM) and arrhythmogenic right ventricular cardiomyopathy (ARVC) are phenotypically distinct inherited cardiac diseases. This case report presents a woman aged 51 years with coinheritance of pathogenic/likely pathogenic variants of the β-myosin heavy chain (*MYH7* p.Glu924Lys) and plakophilin 2 (*PKP2* p.Leu442Argfs∗5), each implicated in HCM and ARVC, respectively. Interestingly, she exhibits the classic HCM phenotype with a heavy arrhythmic burden but no diagnostic features of ARVC. The coinheritance of disease-causing variants in cardiomyopathies has been posited to result in an earlier disease onset and more aggressive clinical course. However, such a relationship has yet to be established when the variants are each robustly associated with different cardiomyopathy phenotypes. The limited existing literature on such cases paints a heterogenous picture of clinical phenotypes with no obvious trend. Here, we explore the interplay between coinheritance of disease-causing variants and resultant disease manifestation, particularly in the context of cardiomyopathies.

## Graphical Abstract

**Figure:**
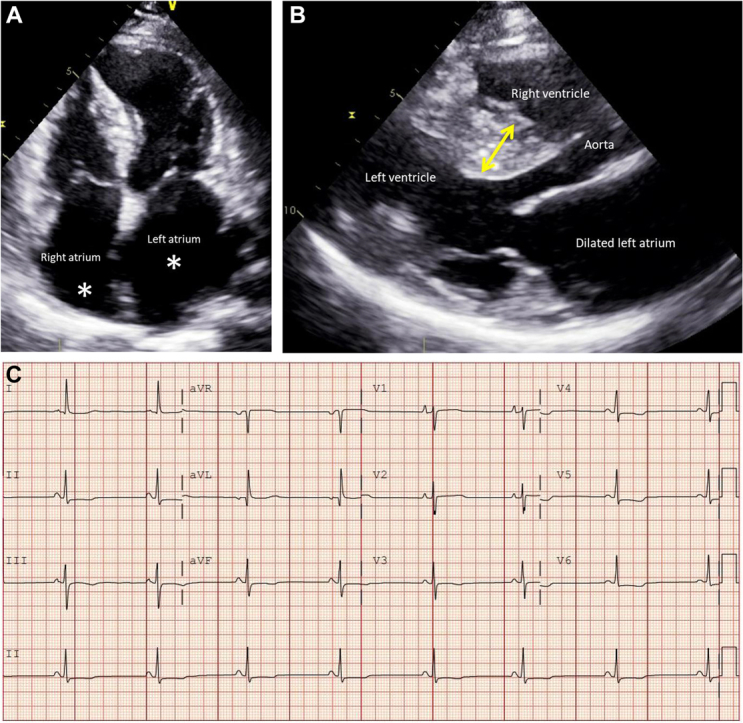

Hypertrophic cardiomyopathy (HCM) is defined by left ventricular (LV) hypertrophy unexplained by abnormal loading conditions, whereas arrhythmogenic right ventricular cardiomyopathy (ARVC) encompasses conditions characterized by right ventricular fibrofatty infiltration, with a predominant arrhythmic presentation. These inherited cardiac diseases are largely attributed to sarcomeric and desmosomal gene variants, respectively.

**Take-Home Messages**

• The coinheritance of multiple distinct cardiomyopathy-associated disease-causing variants is a poorly understood phenomenon with little conclusive evidence on its clinical implications.
• The coinheritance of disease-causing desmosomal variants may contribute to arrhythmic burden in HCM.

The coinheritance of multiple HCM-associated gene variants in phenotypic HCM has an estimated prevalence of 5% and portends earlier disease onset and poorer cardiac and all-cause outcomes.^1^, ^2^, ^3^ However, less is understood about the effect of the coinheritance of multiple disease-causing variants each associated with different cardiomyopathy phenotypes.

We report a patient clinically diagnosed with HCM who displays LV hypertrophy but carries 2 pathogenic/likely pathogenic variants, one of β-myosin heavy chain (*MYH7* p.Glu924Lys), a gene well-established as causative for HCM, and the other of plakophilin 2 (*PKP2* p.Leu442Argfs∗5), the most common desmosomal gene implicated in ARVC. Interestingly, she exhibits no diagnostic features of ARVC. Our report explores the interplay between coinheritance of disease-causing gene variants and resultant disease manifestation.

## History of Presentation

The patient is a 51-year-old Indian woman diagnosed with HCM at age 40 years. She had a heavy arrhythmic burden, with multiple admissions for pre-syncope and dizziness secondary to paroxysmal atrial fibrillation (pAF) with rapid ventricular response (RVR) despite pharmacologic management with bisoprolol, amiodarone, and apixaban.

## Differential Diagnosis

Although the patient experienced numerous bouts of arrhythmias, she did not fulfill the ARVC diagnostic task force criteria. Phenocopies such as glycogen and lysosomal storage disorders were excluded through genetic testing conducted later in her disease course.

## Investigations

**Figure 1:**
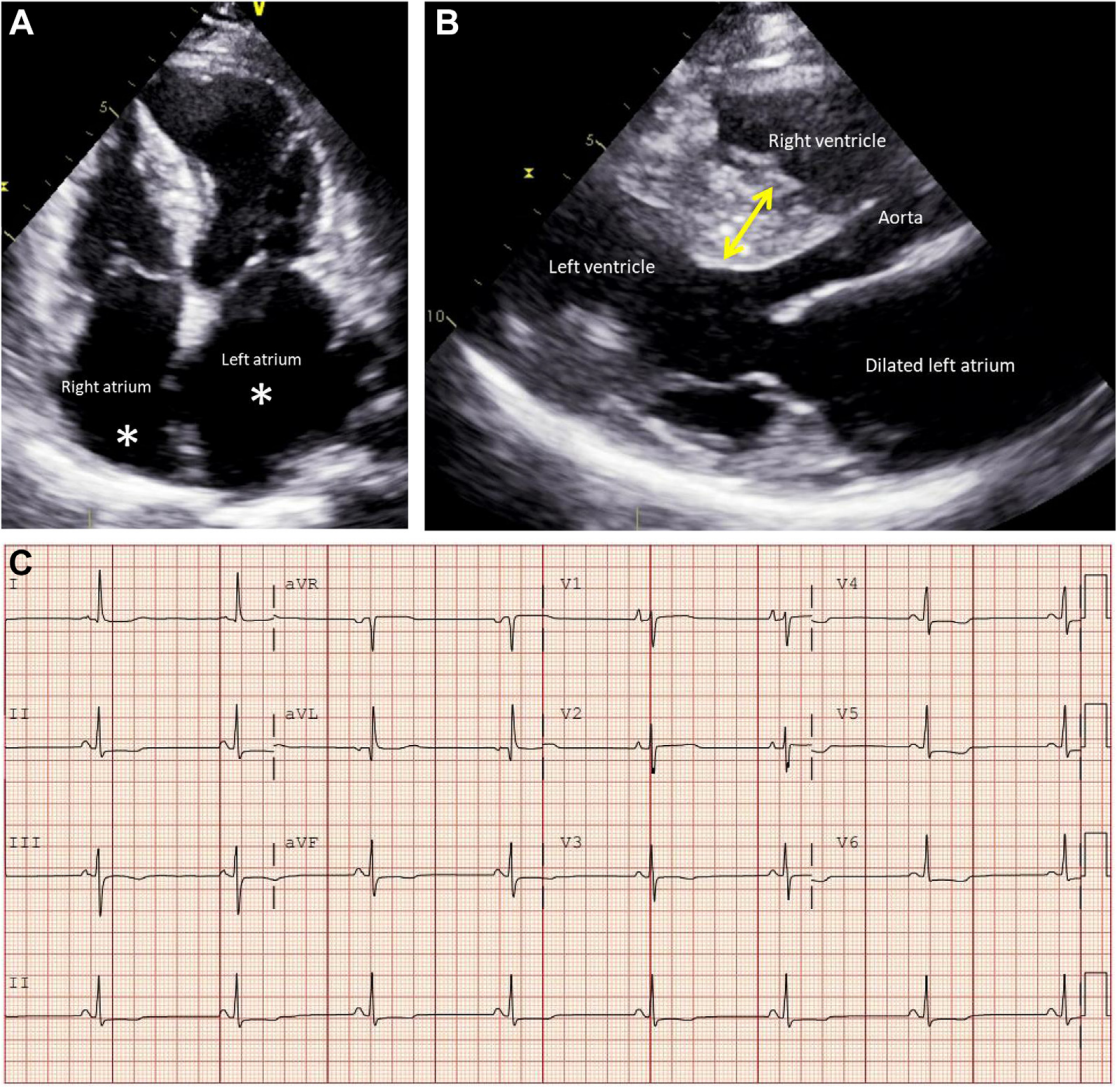
Transthoracic Echocardiogram and Electrocardiogram Results (A) Apical 4-chamber view on transthoracic echocardiogram showing bi-atrial dilatation (∗). (B) Parasternal long-axis view on transthoracic echocardiogram showing asymmetrical LVH involving basal antero-septal LV wall (yellow arrow) with a maximal thickness of 20 mm. (C) Patient’s baseline electrocardiogram showing mild ST-segment depression in septal, lateral and inferior leads. See Videos 1 and 2.

At the point of diagnosis, the patient’s baseline electrocardiogram indicated sinus rhythm with mild ST-segment depression in the septal, lateral, and inferior leads (Figure 1). The transthoracic echocardiogram was notable for severe asymmetrical septal hypertrophy with maximal thickness of 20 mm at the antero-septal LV wall. LV ejection fraction (LVEF) was preserved at 60% with no systolic anterior motion of the mitral valve or LV outflow tract obstruction. Exercise stress echocardiography exhibited no provocable obstruction. She had no valvular pathology or overload conditions such as systemic hypertension to explain the LV hypertrophy.

Nonsustained ventricular tachycardia (NSVT) was noted on Holter monitoring later into the course of her disease, conferring a moderate risk of sudden cardiac death, in the context of HCM. A computed tomography coronary angiogram was done and excluded coronary artery disease.

## Management

Catheter ablation was offered for treatment of pAF with RVR, but the patient initially declined, citing procedural risks. Her disease was further complicated by amiodarone-induced thyrotoxicosis requiring treatment with carbimazole and cessation of amiodarone, and she could not tolerate alternative antiarrhythmic drugs such as sotalol. Eight years’ postdiagnosis, with limited pharmacologic options, she agreed to and underwent a successful pulmonary vein ablation. She was counselled regarding high risk of AF recurrence given severely dilated atria with poor contractile function.

Two years’ postablation, AF with RVR recurred in the setting of septic shock due to gastroenteritis, warranting intensive care unit admission. She then developed hemodynamic compromise requiring synchronized cardioversion. Junctional bradycardia and complete heart block were subsequently noted, necessitating the insertion of a temporary pacing wire.

After resolution of the sepsis, the baseline electrocardiogram showed a low sinus rate suggestive of sinus node dysfunction, a known complication of AF with atrial myopathy. A diagnosis of tachy-brady syndrome was made. The follow-up transthoracic echocardiogram showed a drop in LVEF to 33%, and she eventually underwent insertion of a dual-chamber implantable cardioverter-defibrillator. Heart failure medications were optimized, and she was discharged after cardiac rehabilitation.

## Outcome and Follow-up

Outpatient cardiac magnetic resonance imaging in the study patient revealed improvement in LVEF from 33% to 50%. This change suggested that pAF-mediated, tachycardia-induced cardiomyopathy was the probable etiology of the previous decline in LVEF. Other findings include asymmetrical septal hypertrophy with maximal wall thickness of 18 mm at the antero-septal LV wall and normal right ventricular size with normal right ventricular systolic function and bi-atrial dilatation. Gadolinium was not administered due to significant artifact interference from the implantable cardioverter-defibrillator.

The patient remained well at follow-up with reduction in pAF burden on bisoprolol and digoxin.

As part of the comprehensive work-up for HCM presenting with high arrhythmic burden, the patient was referred to the inherited cardiac genetics clinic for review and testing. The primary objective of this assessment was to ascertain the genetic etiology for her clinical diagnosis of HCM and exclude phenocopies such as glycogen storage disorders (eg, Danon disease) or lysosomal storage disorders (eg, Fabry disease).

**Figure 2:**
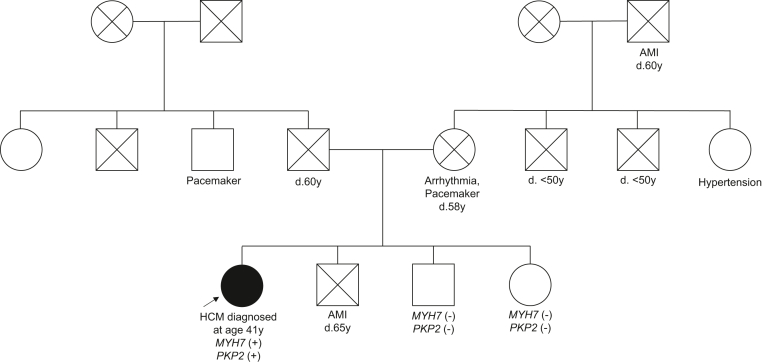
Patient’s Genogram Genogram of the patient containing known phenotypic data of family members is shown. Square box and *circle* represent male and female members respectively. Full circle represents a member diagnosed with hypertrophic cardiomyopathy. Arrow indicates the proband. *Cross symbol* indicates a deceased individual. *d.* indicates age at time of death. *y* indicates age in years. *MYH7* refers to the variant *MYH7* p.(Glu924Lys). *PKP2* refers to the variant *PKP2* p.(Leu442Argfs∗5). AMI = acute myocardial infarction; HCM = hypertrophic cardiomyopathy.

The genogram (Figure 2) illustrates a trend of premature mortality among maternal relatives. A targeted cardiomyopathy gene panel (Table 1) was performed, revealing 2 heterozygous disease-causing variants, *MYH7* (NM_000257.3) c.2770G>A p.Glu924Lys and *PKP2* (NM_004572.3) c.1295_1323dup p.Leu442Argfs∗5, associated with HCM and ARVC, respectively. The *MYH7* variant aligned with the patient’s HCM phenotype, but she did not display any features diagnostic of ARVC.

**Table 1:** Genetic Testing Details and Results

	Variant	Consequence	Zygosity	gnomAD^a^ Global Allele Frequency	ACMG/AMP Criteria	ACMG/AMP Classification
*MYH7*	c.2770G>A p.Glu924Lys	Missense	Heterozygous	Absent	PS3+PS4+PP3	Pathogenic
*PKP2*	c.1295_1323dup p.Leu442Argfs∗5	Loss-of-function	Heterozygous	0.003%	PVS1+PM2	Likely pathogenic

ACMG = American College of Medical Genetics and Genomics; AMP = Association for Molecular Pathology; *MYH7* = β-myosin heavy chain; *PKP2* = plakophilin 2.

a gnomAD version 2.1.1.

Her siblings, who were clinically well, underwent cascade testing and were negative for these 2 disease-causing variants. No other immediate family members were available for cascade testing.

## Discussion

We present a patient with coinheritance of 2 disease-causing variants involved in HCM and ARVC. Her clinical phenotype was HCM attributed to a pathogenic missense variant (*MYH7* p.Glu924Lys) in the *MYH7* gene, 1 of 2 of the most common genes implicated in HCM. Although she also possesses a likely pathogenic loss-of-function variant (*PKP2* p.Leu442Argfs∗5) in the *PKP2* gene, she has not manifested any features of ARVC.

The coinheritance of disease-causing variants in cardiomyopathies is epidemiologically significant, with most studies focused on the digenic inheritance and compound heterozygosity of variants both associated with a specific cardiomyopathy phenotype. Based on current evidence, its prevalence in HCM is up to 5% and has shown association with worse LV hypertrophy and poorer event-free survival from cardiac outcomes and all-cause mortality.^2^^,^^3^ A handful of case reports have also proposed a similar relationship in dilated cardiomyopathy.^4^ In a systematic analysis involving ARVC, coinheritance of pathogenic variants has shown association with earlier onset of ventricular arrhythmias, heart failure, and death.^5^ The existing literature therefore suggests an additive effect of coinheritance, potentially resulting in more aggressive disease courses and poorer clinical outcomes.

However, no clear relationship has been established when the disease-causing variants are each associated with different cardiomyopathy phenotypes. Although data on its prevalence are not yet available, 2 studies have attempted to characterize its clinical significance. A report on 2 families with coinheritance of variants associated with AVRC and HCM found that double heterozygotes displayed variable clinical expression of both cardiomyopathies but not a more severe phenotype than those carrying only one variant.^6^ Another study found that patients diagnosed with HCM possessing deleterious desmosomal variants had a nearly 8-fold greater risk of NSVT than those without.^7^ Although certain associations have been drawn, sufficient evidence is lacking to show a predictable effect of such coinheritance.

The exact implications of the coinheritance found in the current patient are unclear. There is a possibility that the *PKP2* variant was an incidental finding, given that AF and NSVT are known complications of sarcomeric HCM. However, we hypothesize that the patient’s highly arrhythmogenic disease course is potentially attributable to the disease-causing *PKP2* variant. This is having considered its loss-of-function status and the known phenomenon of coinheritance of disease-causing variants in HCM contributing to severe, or at least variable, clinical manifestations.^3^^,^^6^ As discussed, the coinheritance of desmosomal variants in HCM may result in an increased risk of NSVT, although the underlying mechanisms remain undetermined.^7^ *PKP2* variants have also shown association with a higher incidence of NSVT in patients with ARVC.^8^

The absence of overt ARVC features could be explained by incomplete penetrance and variable expressivity, a known feature of adult-onset cardiomyopathies.^9^ In fact, pathogenic variants associated with ARVC, HCM, and dilated cardiomyopathy are not rare in the general population, and carriers exhibit low disease penetrance of around 1.2% to 3.1%.^10^

Although we cannot definitively ascertain whether the disease-causing variants in our patient were de novo or inherited, as both siblings tested negative and no further immediate family members were available for genetic testing, the trend of early mortality among her maternal relatives may offer insights into the potential mode of inheritance. It is possible that her mother, maternal uncles, and maternal grandfather might have also harbored these variants predisposing them to early cardiac death. However, this remains speculative in the absence of medical records and genetic testing.

## Conclusions

Our report aims to contribute to the growing, albeit sparse, body of literature on the coinheritance of multiple distinct cardiomyopathy-associated disease-causing variants. With such coinheritance potentially altering disease trajectory, it is imperative to understand the implications of gene variants for clinicians to better manage patients.

## Funding Support and Author Disclosures

Dr Jamuar is supported by National Medical Research Council Clinician Scientist Award (NMRC/CSAINVJun21-0003). All other authors have reported that they have no relationships relevant to the contents of this paper to disclose.

## Appendix

Video 1 Apical 4-Chamber View on Transthoracic Echocardiogram

Video 2 Parasternal Long-Axis View on Transthoracic Echocardiogram
